# A dated phylogeny of the genus *Pennantia* (Pennantiaceae) based on whole chloroplast genome and nuclear ribosomal 18S–26S repeat region sequences

**DOI:** 10.3897/phytokeys.155.53460

**Published:** 2020-08-07

**Authors:** Kévin J. L. Maurin

**Affiliations:** 1 The University of Waikato – School of Science, Private Bag 3105, Hamilton 3240, New Zealand The University of Waikato Hamilton New Zealand

**Keywords:** chimeric mapping reference, chloroplast DNA, internal transcribed spacer, Next Generation Sequencing, off-target reads, *
Torricellia
*

## Abstract

*Pennantia*, which comprises four species distributed in Australasia, was the subject of a monographic taxonomic treatment based on morphological characters in 2002. When this genus has been included in molecular phylogenies, it has usually been represented by a single species, *P. corymbosa* J.R.Forst. & G.Forst., or occasionally also by *P. cunninghamii* Miers. This study presents the first dated phylogenetic analysis encompassing all species of the genus *Pennantia* and using chloroplast DNA. The nuclear ribosomal 18S–26S repeat region is also investigated, using a chimeric reference sequence against which reads not mapping to the chloroplast genome were aligned. This mapping of off-target reads proved valuable in exploiting otherwise discarded data, but with rather variable success. The trees based on chloroplast DNA and the nuclear markers are congruent but the relationships among the members of the latter are less strongly supported overall, certainly due to the presence of ambiguous characters in the alignment resulting from low coverage. The dated chloroplast DNA phylogeny suggests that *Pennantia* has diversified within the last 20 My, with the lineages represented by *P. baylisiana* (W.R.B.Oliv.) G.T.S.Baylis, *P. endlicheri* Reissek and *P. corymbosa* diversifying within the last 9 My. The analyses presented here also confirm previous molecular work based on the nuclear internal transcribed spacer region showing that *P. baylisiana* and *P. endlicheri*, which were sometimes considered synonyms, are not sister taxa and therefore support their recognition as distinct species.

## Introduction

*Pennantia* J.R.Forst. & G.Forst. is the sole genus of the family Pennantiaceae J.Agardh, a member of Apiales that comprises four species in Australasia (Gardner and de Lange 2002, Fig. 1). *Pennantia endlicheri* Reissek is a forest tree endemic to Norfolk Island, a small volcanic remnant located about 1400 km east of Australia’s mainland. *Pennantia baylisiana* (W.R.B.Oliv.) G.T.S.Baylis (Three Kings Kaikomako/Kaikōmako Manawa Tāwhi) is a small tree originally known in the wild by only one plant, discovered in 1945 on Great Island/Manawa Tawhi (Three Kings Islands/Manawatāwhi, New Zealand, Baylis 1977) and thought to be female. However, cuttings of the plant were induced to produce seeds in cultivation (Beever and Davidson 1999, Gardner et al. 2004) and later the wild individual was observed seeding (Wright 1989). It is nowadays planted throughout New Zealand in both residential and botanic gardens (Gardner and de Lange 2002; pers. obs.) from cuttings of the original tree and from the seeds they produced (de Lange et al. 2010). *Pennantia baylisiana* was regarded by Sleumer (1970) as synonymous with *P. endlicheri*, a view disputed by Baylis (1977, 1989); more recently, Gardner and de Lange (2002) maintained *P. baylisiana* on morphological grounds, while Mabberley (2017) still considered it a synonym of *P. endlicheri*. *Pennantia corymbosa* J.R.Forst. & G.Forst. is a tree endemic to the main islands of New Zealand (North Island, South Island and Stewart Island) and some outlying islands. It is a heteroblastic tree of coastal and lowland forests with a divaricating juvenile form (Dawson and Lucas 2012). *Pennantia cunninghamii* Miers is an Australian endemic tree of subtropical to warm-temperate rainforest of the east coast. Miers (1852) initially placed this species in a monotypic section, P. sect. Dermatocarpus Miers, because of its fruits, which are different from those of *P. corymbosa* and *P. endlicheri*. In Miers’ time, *P. baylisiana* had not yet been collected, and even though it has similar fruits to *P. cunninghamii*, Gardner and de Lange (2002) maintained P. sect. Dermatocarpus on the basis of other morphological traits that distinguish *P. cunninghamii* from the other members of the genus, which they placed in P. sect. Pennantia.

**Figure 1.**
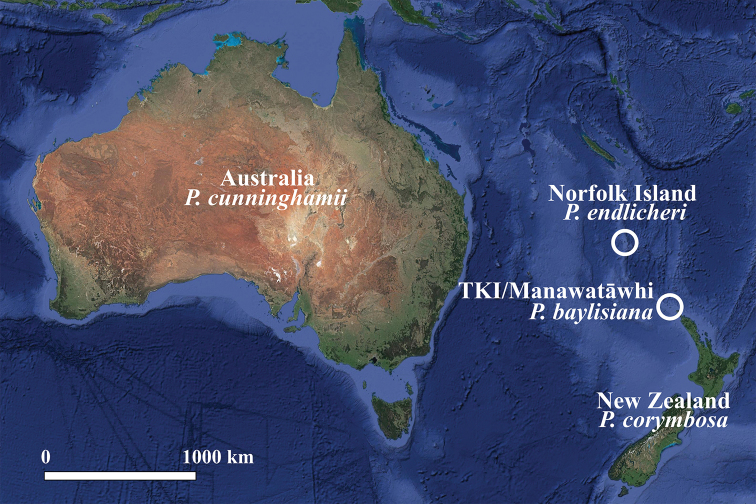
General distribution of the four *Pennantia* species. TKI = Three Kings Islands. Generated in QGIS 3.0.1 from Google Satellite data obtained through the XYZ Tiles tool (https://mt1.google.com/vt/lyrs=s&x={x}&y={y}&z={z}).

The placement of Pennantiaceae within Apiales has been a matter of debate. Their morphology is consistent with Apiales in the inferior position of their ovary and their low number of carpels (Nicolas and Plunkett 2014). On the molecular phylogenetics side, studies have mostly sampled *P. corymbosa* alone (Chandler and Plunkett 2004; Qiu et al. 2010) or with *P. cunninghamii* (Kårehed 2001, 2003; Winkworth et al. 2008; Nicolas 2009; Nicolas and Plunkett 2009, 2014; Tank and Donoghue 2010; Byng et al. 2014; Magallón et al. 2015; Li et al. 2019); Keeling et al. (2004), however, provided a phylogeny of the four species based on the nuclear ribosomal internal transcribed spacer (ITS) region. On one hand, analyses of nuclear markers proved rather ambiguous, sometimes showing that *Pennantia* falls among close sisters to Apiales, namely Dipsacales or Aquifoliales (Chandler and Plunkett 2004; Nicolas 2009), sometimes that it falls among Apiales (Keeling et al. 2004). On the other hand, sequence data from plastid (e.g. Kårehed 2001; Li et al. 2019) and mitochondrial genes (albeit with poor support, Qiu et al. 2010) placed them sister to the rest of the Apiales; this conclusion was strongly supported by studies that built a phylogeny combining both plastid genes and nuclear markers (e.g. Chandler and Plunkett 2004; Magallón et al. 2015).

This study has three goals. (1) To propose the first molecular phylogeny that samples all four species of *Pennantia* for whole plastid DNA sequences, dated using two Apiales fossils and one secondary calibration. (2) To present and evaluate the relevance of a method I used to generate sequence data for nuclear markers at low marginal cost from the shotgun sequencing of genomic DNA: I mapped reads that were unmapped to the chloroplast DNA reference sequence (“off-target reads”) against a chimeric 18S–26S nuclear ribosomal DNA repeat region reference sequence to build the sequences for a nuclear DNA phylogeny. (3) To use both the chloroplast DNA and nuclear DNA phylogenies to further examine proposals made by Gardner and de Lange (2002) regarding the relationships among the four *Pennantia* species based on morphological features alone, which have also been assessed by Keeling et al. (2004) using nuclear ribosomal sequences alone.

## Methods

### Sampling plan

Gardner and de Lange (2002) showed that all four *Pennantia* species are well defined morphologically, and that they have no morphologically divergent populations, a claim which still appears unchallenged today; therefore, it is reasonable in such a group to assume that morphological coherence is an accurate indication of monophyly within each species, and hence only one sample per species was considered. For the chloroplast DNA phylogeny, I also included representatives of five families of Apiales, and four closely related orders according to recent whole-plastid DNA phylogenies of land plants as an outgroup (e.g. Magallón et al. 2015, Li et al. 2019). I included newly generated sequences of the apialean families Araliaceae Juss. (6 species), Pittosporaceae R.Br. (1 species) and Torricelliaceae Hu (2 species), and of the order Asterales (1 species), along with previously published sequences downloaded from GenBank of two other families of Apiales, Apiaceae Lindl. and Torricelliaceae, and of three other orders, Aquifoliales, Dipsacales and Paracryphiales (1 species each); see Table 1 for details. I was not able to generate nor could I find whole-plastid DNA sequences for the remaining two families of Apiales, Griseliniaceae Takht. and Myodocarpaceae Doweld. For the nuclear DNA phylogeny, newly generated sequences of the 18S–26S repeat region for *Pennantia* were obtained from the same samples used to generate the chloroplast DNA sequences. The sequences newly generated for this study were obtained either from field collections that were dried in silica gel and processed in the lab within three months of collection (Maurin collections in Table 1), or from herbarium specimens. I was not able to obtain sequences of the 18S–26S nuclear DNA repeat region from Torricelliaceae. The sampling plan for chloroplast DNA and the 18S–26S nuclear DNA repeat region is given in Table 1. At the time of submission of this paper, a whole chloroplast DNA sequence purported to be of *Torricellia angulata* Oliv. was available on GenBank (accession NC031509/KX648359); it was disregarded because it appears to derive from a member of Rosales. A second *Torricellia* chloroplast genome sequence (NC040944), from *T. tiliifolia* DC., was included.

**Table 1.** Sampling plan of this study, with voucher information and GenBank accession numbers. Samples sorted alphabetically by order name then species name. CANB = Australian National Herbarium (CANB), Canberra, Australia; CHR = Allan Herbarium (CHR), Lincoln, New Zealand; P = Herbarium of the Muséum National d’Histoire Naturelle, Paris, France. Newly generated sequences were formatted for submission to GenBank using the tool GB2Sequin (Lehwark and Greiner 2019).

Order	Family	Species	Distribution	Source of plant material or sequence	Herbarium accession #	Voucher or publication	Markers	GenBank accession #
Apiales	Araliaceae	*Cheirodendron bastardianum* (Decne.) Frodin	Marquesas Islands	P	P02800554	Perlman 19764	Chloroplast	MT385071
	Apiaceae	*Daucus carota* L.	Native to temperate Europe and south-west Asia	GenBank	–	Ruhlman et al. (2006)	Chloroplast	DQ898156
	Torricelliaceae	*Melanophylla alnifolia* Baker	Madagascar	P	P02529054	Ranirison 966	Chloroplast	MT385073
	Torricelliaceae	*Melanophylla modestei* G.E. Schatz, Lowry & A.-E. Wolf	Madagascar	P	P06233571	Bernard 1700	Chloroplast	MT385074
	Pennantiaceae	*Pennantia baylisiana* (W.R.B.Oliv.) G.T.S.Baylis	Three Kings Islands/Manawatāwhi (Great Island/Manawa Tawhi)	CHR	CHR 655088	Maurin 87	Chloroplast	MT385075
							Nuclear	MT434778
				GenBank	–	Rotherdam et al. (unpubl.)	Nuclear	EF660531
	Pennantiaceae	*Pennantia corymbosa* J.R.Forst. & G.Forst.	New Zealand’s main islands and some neighbouring offshore islands	CHR	CHR 649661	Maurin 45	Chloroplast	MT385076
							Nuclear	MT434779
				GenBank	–	Rotherdam et al. (unpubl.)	Nuclear	EF635468
	Pennantiaceae	*Pennantia cunninghamii* Miers	East coast of Australia	CANB	CANB869762	Purdie 9229	Chloroplast	MT385077
							Nuclear	MT434780
				GenBank	–	Rotherdam et al. (unpubl.)	Nuclear	EF635470
	Pennantiaceae	*Pennantia endlicheri* Reissek	Norfolk Island	CANB	CBG8703383	Telford 10450	Chloroplast	MT385078
							Nuclear	MT434781
				GenBank	–	Rotherdam et al. (unpubl.)	Nuclear	EF635469
	Pittosporaceae	*Pittosporum eugenioides* A.Cunn.	North and South Islands of New Zealand	CHR	CHR 553618	Courtney, *s.n.*	Chloroplast	MT385079
	Araliaceae	*Raukaua anomalus* (Hook.) A.D.Mitch., Frodin & Heads	New Zealand’s main islands	CHR	CHR 649673	Maurin 57	Chloroplast	MT385080
	Araliaceae	*Raukaua edgerleyi* (Hook.f.) Seem.	New Zealand’s main islands	CHR	CHR 655508	Maurin 103	Chloroplast	MT385081
	Araliaceae	*Raukaua simplex* (G.Forst.) A.D.Mitch., Frodin & Heads	New Zealand’s main islands, Auckland Islands	CHR	CHR 437312	Sykes 42/87	Chloroplast	MT385082
	Araliaceae	*Schefflera actinophylla* (Endl.) Harms	Northern and north-eastern coast of Australia	CANB	CANB874342	Lepschi 7083	Chloroplast	MT385083
	Araliaceae	*Schefflera digitata* J.R.Forst. & G.Forst.	New Zealand’s main islands	CHR	CHR 649676	Maurin 60	Chloroplast	MT385084
	Torricelliaceae	*Torricellia tiliifolia* DC.	China, eastern Himalaya	GenBank	–	Yao et al. (2019)	Chloroplast	NC040944
Aquifoliales	Aquifoliaceae	*Ilex paraguariensis* A.St.-Hil.	South America	GenBank	–	Cascales et al. (2017)	Chloroplast	KP016928
Asterales	Argophyllaceae	*Corokia cotoneaster* Raoul	New Zealand’s main islands	CHR	CHR 655097	Maurin 96	Chloroplast	MT385072
Dipsacales	Caprifoliaceae	*Dipsacus asper* Wallich ex Candolle	China, south-east Asia	GenBank	–	Park et al. (2018)	Chloroplast	MH074864
Paracryphiales	Paracryphiaceae	*Quintinia verdonii* F.Muell.	Eastern Australia	GenBank	–	Yao et al. (2019)	Chloroplast	MK397891

### DNA extraction

DNA from the samples of *Pennantia corymbosa*, *Raukaua anomalus* (Hook.) A.D.Mitch., Frodin & Heads and *Schefflera digitata* J.R.Forst. & G.Forst. was extracted using a CTAB-based protocol (Doyle and Dickson 1987) modified as in Smissen and Heenan (2007) to include a phenol:chloroform extraction and recovery using spin columns (Zymo IIC, Zymo Research, Orange County, California). The DNA of the other samples was extracted following the DNA tissue protocol of the Maxwell 16 instrument (Promega, Madison, Wisconsin) and further purified by phenol/chloroform extraction and recovery in spin columns. Detailed step-by-step protocols are available upon request. The DNA concentration of the extracts was measured using the Qubit (Thermo Fisher Scientific, Waltham, Massachusetts) dsDNA high-sensitivity assay protocol.

### Library preparation and sequencing

Genomic DNA libraries of *Pennantia corymbosa*, *Raukaua anomalus* and *Schefflera digitata* were prepared using Illumina Nextera DNA Library Prep kits, following the manufacturer’s instructions (Reference Guide, #15027987 v01, January 2016) except that I halved the quantities of reagents and the target amount of input DNA. Libraries of the other samples were prepared using Illumina TruSeq Nano DNA Library Prep kits, according to the manufacturer’s instructions (Reference Guide, # 15041110 Rev. D, June 2015), again using halved reagent quantities and target input DNA; genomic DNA was fragmented using a Covaris ME220 Focused-ultrasonicator (settings: 75 s duration – 40 W peak power – 25% duty factor – 50 cycles per burst). The concentration and size range of libraries were measured with a LabChip GX Touch HT (Perkin Elmer). Libraries were enriched for chloroplast DNA using a custom MYBaits kit (Arbor Biosciences, Ann Arbor) modified from Stull et al. (2013) as detailed in Smissen et al. (in press) using the manufacturer’s instructions (version 3.02, July 2016 or version 4.01, April 2018). Illumina HiSeq shotgun sequencing was carried out by Otago Genomics using paired end 2 × 125 bp reads.

### Chloroplast DNA assembly and annotation

Reads were first trimmed using Trimmomatic v. 0.38 (Bolger et al. 2014) with the following settings: ILLUMINACLIP:[path/to/NexteraPE-PE.fa for *Pennantia corymbosa*, *Raukaua anomalus* and *Schefflera digitata*, TruSeq3-PE-2.fa for the others]:1:30:10 SLIDINGWINDOW:10:20 MINLEN:40. The reads of the Pennantiaceae and Torricelliaceae samples were then mapped to *Torricellia tiliifolia* (NC040944), the closest sequence to *Pennantia* available in GenBank at the time the mappings were performed (July 2019) that was both verified and published. Mapping was performed with BWA, using the BWA-MEM algorithm (Li 2013). The quality of the best resulting sequence, *P. cunninghamii*, was then improved (in terms of coverage, HQ% and number of ambiguous bases) by remapping its reads against a consensus sequence from the initial mapping against the *Torricellia* sequence. Finally, reads from all the other samples were mapped against the remapped *P. cunninghamii* sequence. The same process was followed for Araliaceae with the sequence of *Schefflera actinophylla* (Endl.) Harms, first mapped to the GenBank reference *Schefflera heptaphylla* (L.) Frodin (NC029764), *Pittosporum eugenioides* A.Cunn. first mapped to *Torricellia tiliifolia* (NC040944), and *Corokia cotoneaster* Raoul first mapped to *Llerasia caucana* (S.F.Blake) Cuatrec. (NC034821).

The resulting sequences, except *Melanophylla modestei* G.E. Schatz, Lowry & A.-E. Wolf, were of good overall quality (Suppl. material 1: Table S1): on average the HQ% was 98.4 (range: 93.7 – 99.9) and the percentage of ambiguous bases was 1.2% (range: 0.2% – 6.2%). Mean coverage ranged from 124 to 10,804. The *Melanophylla modestei* sequence was of lesser quality, with HQ% 63.6 and mean coverage of 16.5. However, its percentage of ambiguous bases was still low (4.8%), with the vast majority of them located outside the coding regions used in the phylogenetic analysis. The sequences were annotated by (1) aligning the improved references to the GenBank references used to map their reads against with the MAFFT algorithm v. 7.388 (Katoh et al. 2002, Katoh and Standley 2013) plugin in Geneious Prime 2019.2.1, (2) transferring the annotations of the GenBank references to the improved references, and (3) aligning the other sequences to their corresponding improved references, again with MAFFT within Geneious Prime, and transferring the annotations across. Annotations were manually checked.

### 18S–26S nuclear ribosomal DNA repeat region assembly and annotation

In the absence of a complete 18S–26S nuclear ribosomal DNA repeat region for Apiales, I built a chimeric 18S–26S nuclear DNA repeat region from several GenBank sequences. I concatenated the 18S rRNA sequence of *Melanophylla alnifolia* Baker (AJ236002), the ITS1, 5.8 S RNA, and ITS2 sequences of *Pennantia cunninghamii* (EF635470), and the 26S rRNA sequence of *Pittosporum fairchildii* Cheeseman (AF479192), in that order. The structure of the resulting chimeric 18S–26S nuclear DNA repeat region is provided in Suppl. material 1: Fig. S1. I then mapped the off-target reads from the chloroplast DNA mappings of the shotgun sequencing data of my herbarium and fresh samples to this chimeric nuclear DNA reference.

The quality of the resulting assemblies was rather variable. There was no clear relationship between the number of reads available to map and the number of reads actually mapped to the chimeric reference (Suppl. material 1: Table S2). The mapping of the two *Melanophylla* species failed; the mapping of the four sequences of *Pennantia* was satisfactory for *P. baylisiana*, *P. cunninghamii* and *P. endlicheri* (HQ% > 86% and ambiguities < 7%), but less so for *P. corymbosa* (HQ% = 51.0%, and ambiguities = 29.1%). Because of the variable quality of my newly reconstructed 18S–26S nuclear DNA repeat region sequences, I aligned them together with the longest sequences of the 18S–26S nuclear DNA repeat region available on GenBank for the four *Pennantia* species, as a control of the identity of my newly generated sequences for the phylogenetic analyses. Some statistics regarding these sequences discussed later in the paper were obtained with MEGA X (Kumar et al. 2018).

### Data partitioning

Sixty protein-coding sequences (CDS, 46,051 sites) from the long and short single copy regions were used for the chloroplast DNA analyses (see list in Suppl. material 1: Table S3); coding rRNA, which was located in the inverted repeats, was not considered. CDS were partitioned into 1^st^ + 2^nd^ codon position on the one hand (30,701 sites), and 3^rd^ codon position on the other hand (15,350 sites). For the nuclear DNA analyses, the 18S–26S nuclear DNA repeat region alignment represented 810 sites, partitioned as ITS1 + ITS2 on the one hand (538 sites), a portion of 18S rRNA + whole 5.8S rRNA + a portion of 26S rRNA on the other hand (272 sites). The markers were aligned in Geneious Prime using the MAFFT plugin, and the alignments were manually checked.

### Phylogenetic analyses and chloroplast DNA tree calibration

Phylogenetic analyses were conducted with the BEAST suite v. 2.5.2 (Bouckaert et al. 2019). Each of the four partitions was assigned its own evolutionary model using bModelTest (Bouckaert and Drummond 2017) to average the best-fitted nucleotide models. A relaxed clock with rates drawn from an exponential distribution (Drummond et al. 2006) was associated to each partition. The MCMC chains were run for 250 million generations and sampled once every 25,000 generations for chloroplast DNA, and for 50 million generations sampled once every 5,000 generations for nuclear DNA. The influence of tree prior choice on the phylogeny and dating was assessed by repeating the analysis under both the Yule model (Yule 1925) and the Birth-Death model (Gernhard 2008). These analyses were run on the CIPRES platform (Miller et al. 2010). The proper convergence of the chains and the determination of the burnin that would maximise their effective sample size (ESS) was examined with Tracer v. 1.7.1 (Rambaut et al. 2018); the ESS of a parameter represents the number of effectively independent samples from the posterior distribution of the parameter, and therefore how strong its estimation is: values above 200 are considered satisfactory (BEAST Developers 2017). Three independent runs per analysis (i.e. per combination of Birth-Death or Yule model with chloroplast DNA or nuclear DNA) were started from different seeds and combined with LogCombiner v. 2.5.2 (Bouckaert et al. 2019). The combined sampled trees from each analysis were then summarised in TreeAnnotator v. 2.5.2 (Bouckaert et al. 2019) with their selected burnin.

The chloroplast DNA phylogeny was calibrated using two fossils and one secondary calibration. Firstly, I assigned the age of the earliest confirmed fossils of *Torricellia*, which are ca. 48 My old (Manchester et al. 2017), to the minimum crown age of Torricelliaceae, using an offset exponential distribution (Mean = 20.0, Offset = 48.0), resulting in a wide prior with a 2.5% quantile of 48.5 My, a 97.5% quantile of 122 My, and a mean of 68 My. Secondly, I assigned the age of *Paleopanax oregonensis* Manchester fossils, which are considered from the Middle Eocene (Manchester 1994), to the minimum crown age of Araliaceae, following Magallón et al. (2015) and Li et al. (2019); I used an offset exponential distribution (Mean = 20.0 and Offset = 37.8), resulting in a wide prior with a 2.5% quantile of 38.3 My, a 97.5% quantile of 112 My, and a mean of 57.8 My. Finally, the estimated age of Apiales in recent Angiosperm-wide phylogenies (Magallón et al. 2015, Li et al. 2019) is about 80–81 My old, with a maximum interval of about [70,95] My; I therefore assigned an offset lognormal distribution with M = 33.0, S = 0.2, and Offset = 48.0 to the crown age of the Apiales species, resulting in a prior with a 2.5% quantile of 69.9 My, a 97.5% quantile of 95.9 My, and a mean of 81.0 My.

The robustness of the Bayesian inference of tree topology for the phylogenies resulting from both the chloroplast DNA and the nuclear DNA sequence data was assessed with a maximum likelihood approach. RAxML v. 8.2.12 (Stamatakis 2014) was run on CIPRES with the following settings for both phylogenies: GTRGAMMA model, rapid bootstrap analysis with search for best scoring tree (-f a -x) with 1,000 bootstrap replicates. The chloroplast DNA phylogeny was rooted by fixing the four non-Apiales sequences as outgroups, while no outgroup was set for the nuclear DNA phylogeny.

Finally, the six resulting trees (chloroplast DNA or nuclear DNA, with BEAST2/Birth-Death model, BEAST2/Yule model or RAxML) were first formatted in FigTree v. 1.4.4 (Rambaut 2018) and then refined in Inkscape v. 0.92.3. Given the much larger number of sites in the chloroplast DNA dataset compared to the nuclear DNA dataset, a combined analysis was not conducted as its results would have been skewed towards what was observed with chloroplast DNA alone; moreover, the topologies of both phylogenies were congruent. The detailed settings and parameters used for the phylogenetic analyses are in the BEAST2 and RAxML files provided in Suppl. material 2.

## Results

### Dated chloroplast DNA phylogeny

**Figure 2.**
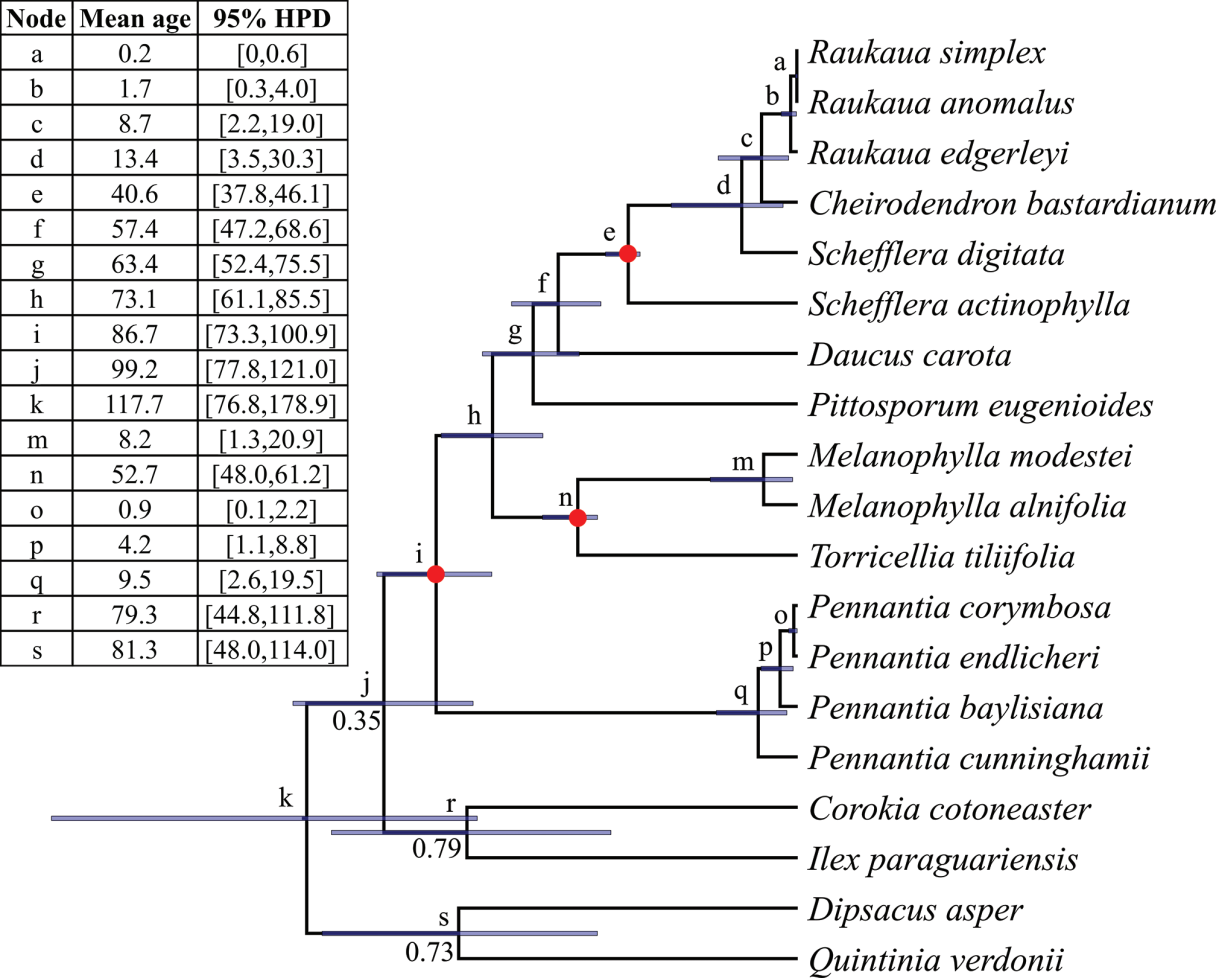
Dated chloroplast DNA BEAST 2 phylogeny of *Pennantia*, under the Birth-Death model. Mean node age and 95% HPD (in My) is given in the table embedded in the figure under the corresponding letter code. 95% HPD is also represented by blue bars. All node posterior probabilities are equal to 1 except if indicated otherwise. The calibrated nodes (see text) are indicated by red dots.

The combination of the chains run under the Birth-Death model or the Yule model resulted in an Effective Sample Size (ESS) > 200 for all their parameters. The tree had the same topology and was very well supported within the ingroup Apiales under both models, all the node posterior probabilities (PP) being equal to 1. Moreover, the same topology was obtained for the chloroplast DNA tree built with RAxML, with 100% bootstrap support within Apiales. The tree resulting from the Birth-Death model is shown in Fig. 2, and the trees resulting from the Yule model and the RAxML analysis in Suppl. material 1: Fig. S2 and Fig. S3 respectively.

In the phylogeny presented in Fig. 2, the relationships between the families of Apiales that were included in the analysis conformed to contemporary ideas about the relationships among Apiales families (Stevens 2017). Here, the crown age of *Pennantia* was estimated at 9.5 My, with an HPD of [2.6,19.5] My. Within *Pennantia*, the Australian species *P. cunninghamii* was sister to the rest of the genus. Then, *P. baylisiana*, from the Three Kings Islands/Manawatāwhi, was sister to a clade formed by the New Zealand species *P. corymbosa* and the Norfolk Island species *P. endlicheri*.

### Undated 18S–26S nuclear DNA repeat region phylogeny

**Figure 3.**
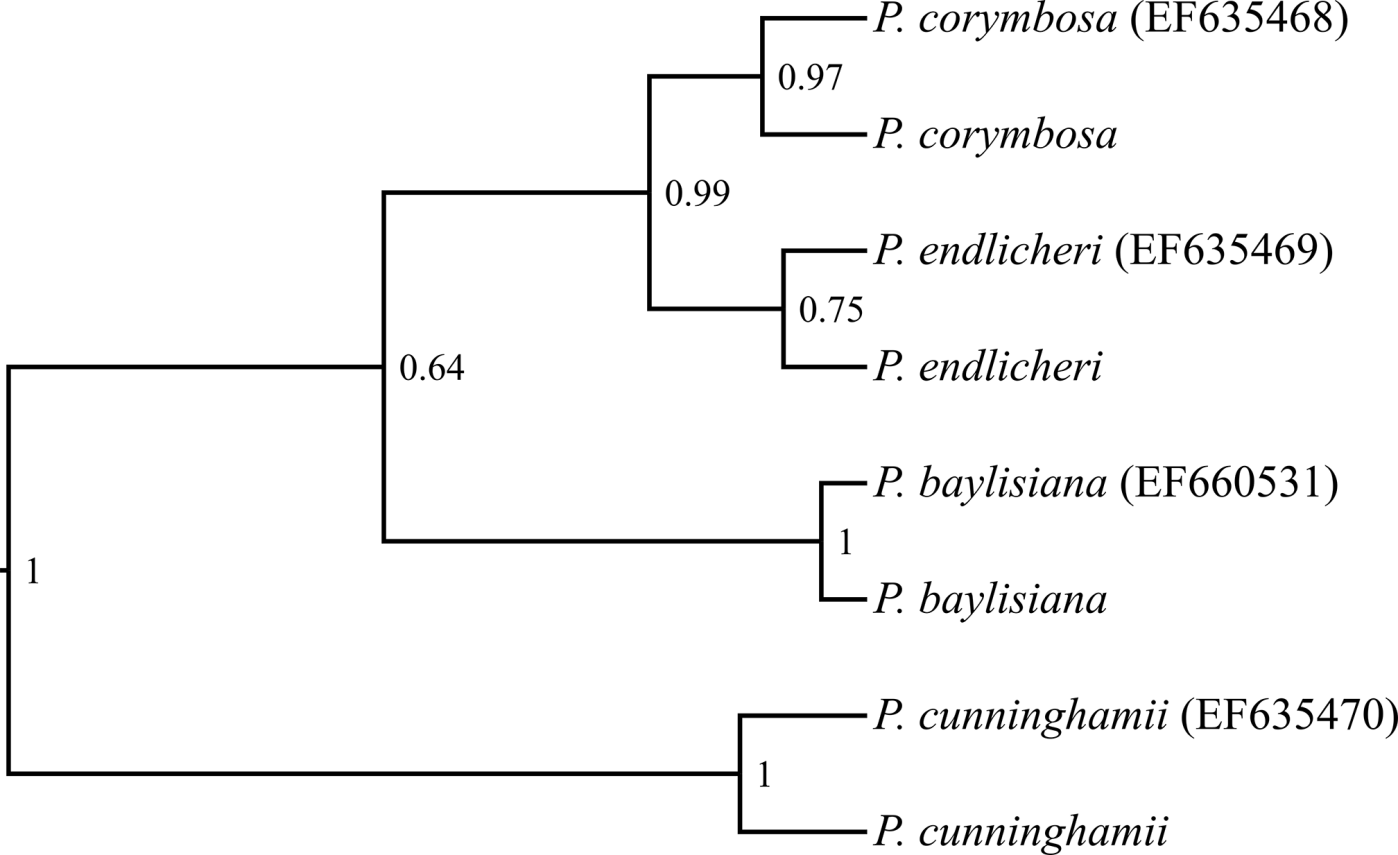
Undated 18S–26S nuclear DNA repeat region BEAST 2 phylogeny of *Pennantia*, under the Birth-Death model. The tree was rooted to make *P. cunninghamii* sister to the other species of *Pennantia*, in accordance with the chloroplast DNA tree and the ITS tree of Keeling et al. (2004). Node posterior probability is shown next to the corresponding node. The sequences downloaded from GenBank have their accession number in round brackets; the others were generated from the samples used in this study.

The chains yielded an ESS far greater than 200 even before they were combined under both the Birth-Death model and the Yule model. The resulting tree showed the same topology with comparable PP under both models, although the PP under the Yule model tended to be slightly lower than under the Birth-Death model. The topology of the tree produced from the RAxML analysis was congruent with the topology of the BEAST2 trees, with bootstrap values of 100% except for the node placing the two samples of *P. corymbosa* and *P. endlicheri* as sister to each other (bootstrap = 88%). For consistency with the chloroplast DNA phylogeny, I draw conclusions regarding the nuclear DNA phylogeny primarily by examining the Birth-Death model tree (Fig. 3), while providing the Yule model and RAxML trees in Suppl. material 1: Fig. S4 and Fig. S5 respectively. In the absence of suitable outgroup sequences, the RAxML nuclear DNA tree was rooted to make *P. cunninghamii* sister to the other species of *Pennantia*, in accordance with the topology of the chloroplast DNA tree presented in this study and of the ITS tree of Keeling et al. (2004).

The percentage of identical sites between the two samples of each species was ≥ 98.7%. There were relatively few parsimony-informative sites in the nuclear DNA alignment: only 35 out of 538 (6.5%) sites in the ITS1/ITS2 partition and 0 out of 272 in the rRNA partition. The two samples of each species were recovered as sisters, usually with strong support: PP = 1 for *P. cunninghamii* and *P. baylisiana*, PP = 0.97 for *P. corymbosa*, but PP = 0.75 only for *P. endlicheri*. Moreover, the topology of this tree was congruent with that of the tree based on chloroplast DNA (Fig. 2), with strong support (PP = 0.99) for the clade *P. corymbosa* + *P. endlicheri* but weak support for the clade *P. corymbosa* + *P. endlicheri* + *P. baylisiana* (PP = 0.64), although the latter had 100% bootstrap support in the RAxML analysis. This phylogeny was also congruent with the one reported by Keeling et al. (2004), built with the maximum likelihood option of PAUP* 4.0b (Swofford 2002), and showing comparable bootstrap values for equivalent nodes in the case of the present RAxML analysis.

## Discussion

### Congruence between chloroplast and nuclear DNA phylogenies

Phylogenies based on chloroplast DNA markers and the 18S–26S nuclear DNA repeat region indicate the same relationships among the four species of *Pennantia*. They are also congruent with the ITS phylogeny of Keeling et al. (2004), confirming the relationships they inferred among the four species. The relatively low support values that were observed for some clades in the 18S–26S nuclear DNA repeat region could result from the limited amount of variation of this region, or more probably from the loss of sites during the phylogenetic analyses due to the presence of ambiguities: the sequences I generated from the samples of *P. endlicheri* and *P. corymbosa* have a percentage of ambiguities of 16.3% and 6.9% respectively (while all the other sequences have ≤ 0.5% of ambiguities), after trimming the sequences. Conflicting tree topologies did not seem to be in play in this case given the paucity of parsimony-informative sites in this 18S–26S nuclear DNA repeat region.

### Crown age of Pennantiaceae and age of its most recent common ancestor (MRCA) with Torricelliaceae

The age of the MRCA of Pennantiaceae and Torricelliaceae (which is the crown age of Apiales) was estimated about 86.7 My, with an HPD of [73.3,100.9] My. This mean estimate is consistent with some of the previous dated phylogenies that include this MRCA: 73.6 My (Li et al. 2019), 80.8 My (Magallón et al. 2015) and 91.39 My (Tank et al. 2015); however, it is more recent than the 117.0 My indicated by Nicolas and Plunkett (2014), which might be explained by their use of an Araliaceae fossil about the same age as the one I used to date a node that is internal to Araliaceae.

The mean crown age of Pennantiaceae was estimated to be 9.5 My with an HPD of [2.6,19.5] My, which is slightly older than the previous estimate for *Pennantia* of 6.6 My with an HPD of ca. [1.6,15.8] My suggested by Nicolas and Plunkett (2014). The fact that I used more conservative priors than they did for the MRCAs of Araliaceae and Torricelliaceae may explain my older estimates. The difference in priors on the crown age of Araliaceae was mentioned above. Moreover, their priors were tightly constrained around old ages compared to mine, e.g. for the crown age of Torricelliaceae they used a prior with a 95% HPD of [55.8,58.7] My, while my prior had a 95% HPD of [48.5,122] My. I allowed the possibility for relatively older posterior dates than the estimated age of the fossils so as to account better for the fact that fossils can only represent the youngest possible age of the clade to which they are associated; older fossils might yet exist and be discovered. Nevertheless, the results of both sets of analyses suggest that *Pennantia* diversified within the last 20 My. The present analysis also shows that the diversification of the ancestors of the extant New Zealand, Three Kings Islands/Manawatāwhi and Norfolk Island species is much more recent, starting about 4.2 Mya with an HPD of [1.1,8.8] My.

### Relationships within *Pennantia*

The phylogenies presented here significantly supported *Pennantia baylisiana* being a distinct species to *Pennantia endlicheri*, corroborating the conclusions Keeling et al. (2004) made from their ITS region phylogeny of the four species of *Pennantia*. Gardner and de Lange (2002) suggested that the closest relative of *P. baylisiana* may be *P. endlicheri* (p. 671) but maintained *P. baylisiana* distinct from *P. endlicheri* on morphological grounds: e.g. domatia developed and bearing trichomes in the former but hardly developed and glabrous in the latter. The chloroplast DNA phylogeny strongly supported the distinction between these species since they are not sister taxa, as it placed *P. baylisiana* sister to the clade *P. endlicheri* + *P. corymbosa* with a PP of 1. In the nuclear DNA phylogeny, this node only had a PP of 0.64 but is strongly supported (bootstrap = 100%) in the phylogeny of Keeling et al. (2004). Characters shared between *P. endlicheri* and *P. corymbosa* that are not found in the two other species of the genus include the presence of uncinate trichomes (rather sparse and restricted to inflorescence axes in *P. endlicheri*) and a stigmatic ring being made of three distinct stigmas (Gardner and de Lange 2002).

The present phylogenies also supported the placement by Miers (1852) of *Pennantia cunninghamii* in a monotypic section Dermatocarpus, which was maintained by Gardner and de Lange (2002) on morphological grounds. *P. cunninghamii* indeed has unique morphological features compared to the rest of the genus. For example, its domatia form pits while those of the other species are pockets (although shallow and sometimes absent in *P. endlicheri*), and its ovary is longitudinally ridged and thus appears to be formed by three carpels while the ovary of the other species is barrel-shaped and barely furrowed. Here, the results of the phylogeny based on chloroplast sequences were consistent with this infrageneric classification, placing *P. cunninghamii* sister to all the other *Pennantia* species with a posterior probability of 1. The nuclear DNA phylogeny presented here, in the absence of outgroups to *Pennantia*, does not explicitly support this idea, but it is consistent with it. The sister group relationship between *P. cunninghamii* and the rest of the genus was well supported by the ITS phylogeny of Keeling et al. (2004, bootstrap values ≥ 96%).

## Conclusions

The analysis of chloroplast genome sequences supports previous phylogenetic results based on nuclear DNA in suggesting that *Pennantia cunninghamii* is sister to the rest of the genus. Moreover, it strongly supports previous nuclear DNA analyses in placing *P. baylisiana* as sister to the clade *P. endlicheri* + *P. corymbosa* rather than sister to *P. endlicheri* alone, with which it has sometimes been considered conspecific (e.g. Mabberley 2017). This is consistent with previous studies based on morphology, which concluded that *P. baylisiana* should be recognised as a distinct species. The dated phylogeny presented here suggests that *Pennantia* diversified within the last 20 My, and possibly as recently as 2.6 My ago. It also suggests that divergences among the ancestors of the three species of section Pennantia, now distributed on Norfolk Island, Three Kings Islands/Manawatāwhi and the main islands of New Zealand, happened over the last 9 My and as recently as 0.1 My ago. However, the island endemism of each *Pennantia* species and the lack of close outgroups and of information about ancestral distribution areas prevents the inference of confident biogeographical scenarios regarding the origin of the distribution of the extant species. Finally, this study has shown that the use of a chimeric reference sequence to utilise off-target reads from target enrichment libraries that are usually discarded can provide useful data for phylogenetic analysis. Although the quality of such mappings can be quite variable, as demonstrated here, the low marginal cost of this procedure makes it worth exploring in genome-based research using shotgun sequencing techniques.

## Supplementary materials

Supplementary material 1 Figs S1–S5; Tables S1–S3 Data type figures and tables https://binary.pensoft.net/file/440093This dataset is made available under the Open Database License (http://opendatacommons.org/licenses/odbl/1.0/). The Open Database License (ODbL) is a license agreement intended to allow users to freely share, modify, and use this Dataset while maintaining this same freedom for others, provided that the original source and author(s) are credited.Author: Kévin J. L. Maurin

Supplementary material 2 BEAST2 and RAxML files Data type molecular data https://binary.pensoft.net/file/440094This dataset is made available under the Open Database License (http://opendatacommons.org/licenses/odbl/1.0/). The Open Database License (ODbL) is a license agreement intended to allow users to freely share, modify, and use this Dataset while maintaining this same freedom for others, provided that the original source and author(s) are credited.Author: Kévin J. L. Maurin
