# Supplementary material for: A dated phylogeny of the genus Pennantia (Pennantiaceae) based on whole chloroplast genome and nuclear ribosomal 18S–26S repeat region sequences
Source: PhytoKeys. 2020 Aug 7;155:15–32. doi: 10.3897/phytokeys.155.53460 (PMC7428460; doi:10.3897/phytokeys.155.53460)
Supplement: Supplementary material 1 — Figs S1–S5; Tables S1–S3 [file phytokeys-155-015-s001.docx]

**A dated phylogeny of the genus *Pennantia* (Pennantiaceae) based on whole chloroplast genome and nuclear ribosomal 18S-26S repeat region sequences – Supplementary Material 1**

KÉVIN J. L. MAURIN

**Supplementary Figure 1.** Structure of the chimeric reference of the 18S–26S nuclear DNA repeat region against which were mapped the reads that were not mapped to the chloroplast DNA reference.

| 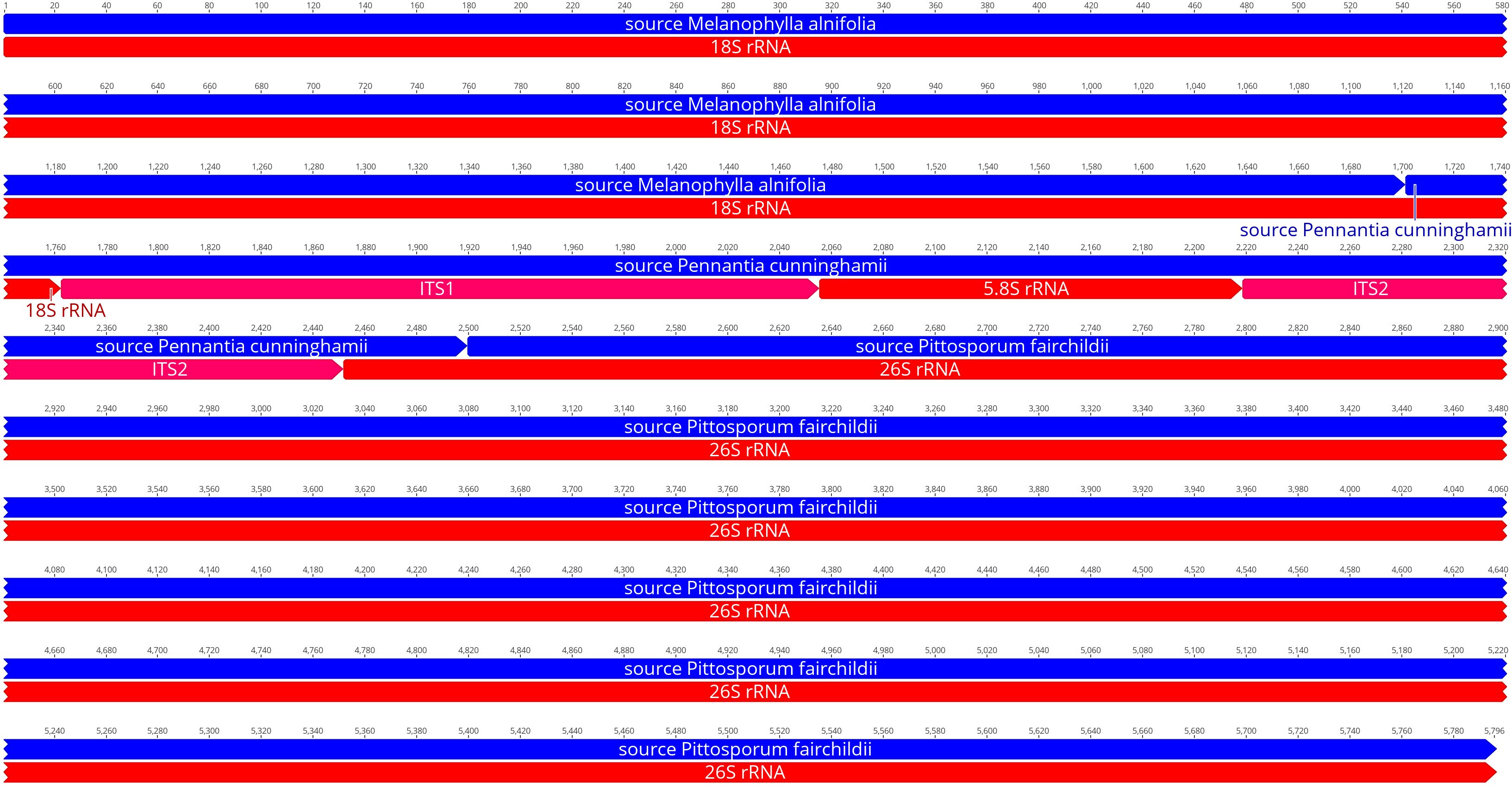 |
| --- |

**Supplementary Figure 2.** Dated chloroplast DNA BEAST 2 phylogeny of *Pennantia*, under the Yule model. Mean node age and 95% HPD (in My) is given in the table embedded in the figure under the corresponding letter code. 95% HPD is also represented by blue bars. All node posterior probabilities are equal to 1 except if indicated otherwise. The calibrated nodes (see text) are indicated by red dots.

| 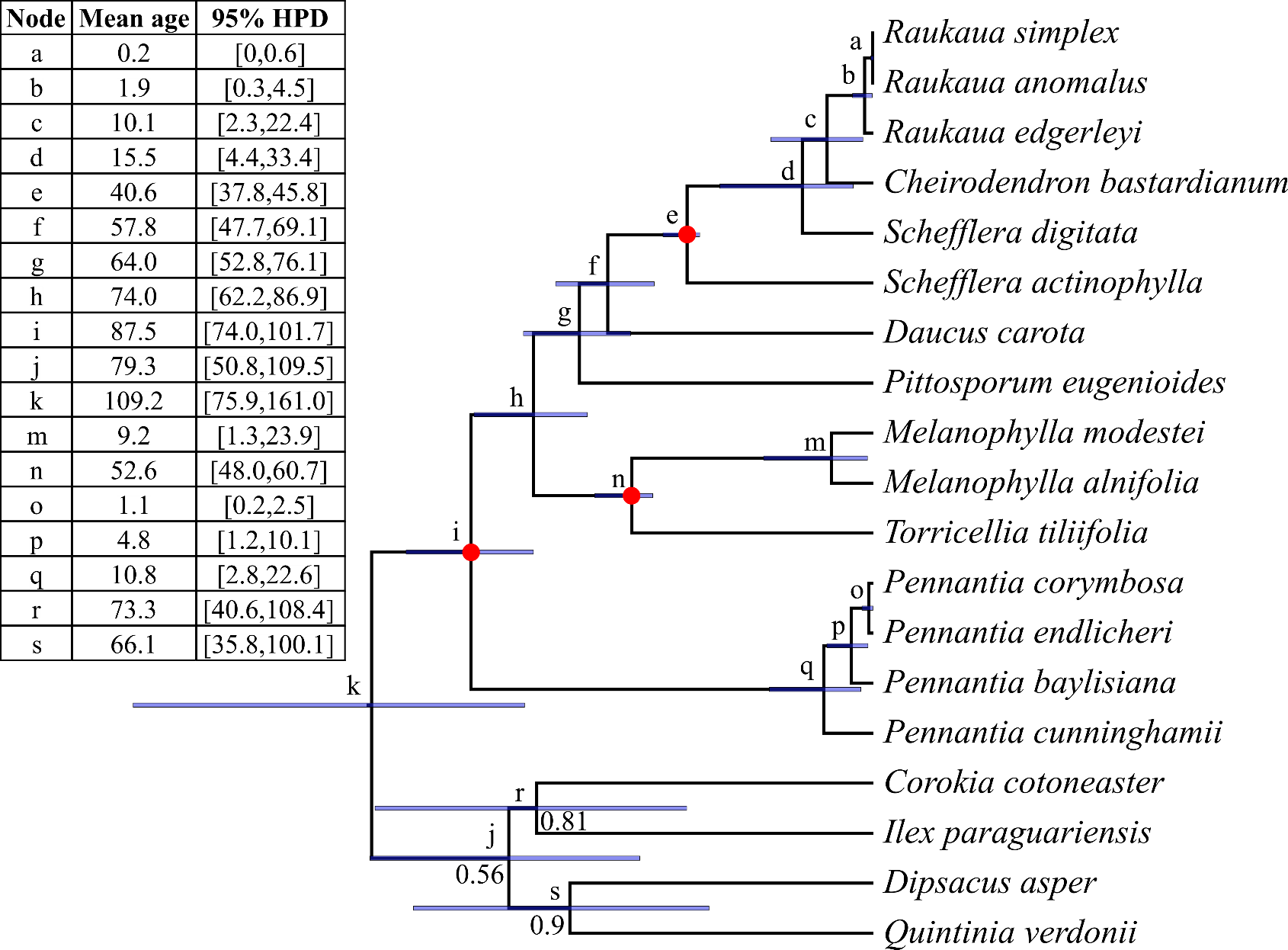 |
| --- |

**Supplementary Figure 3.** RAxML reconstruction of the chloroplast DNA phylogeny of *Pennantia*. Bootstrap value is indicated next to the corresponding node.

| 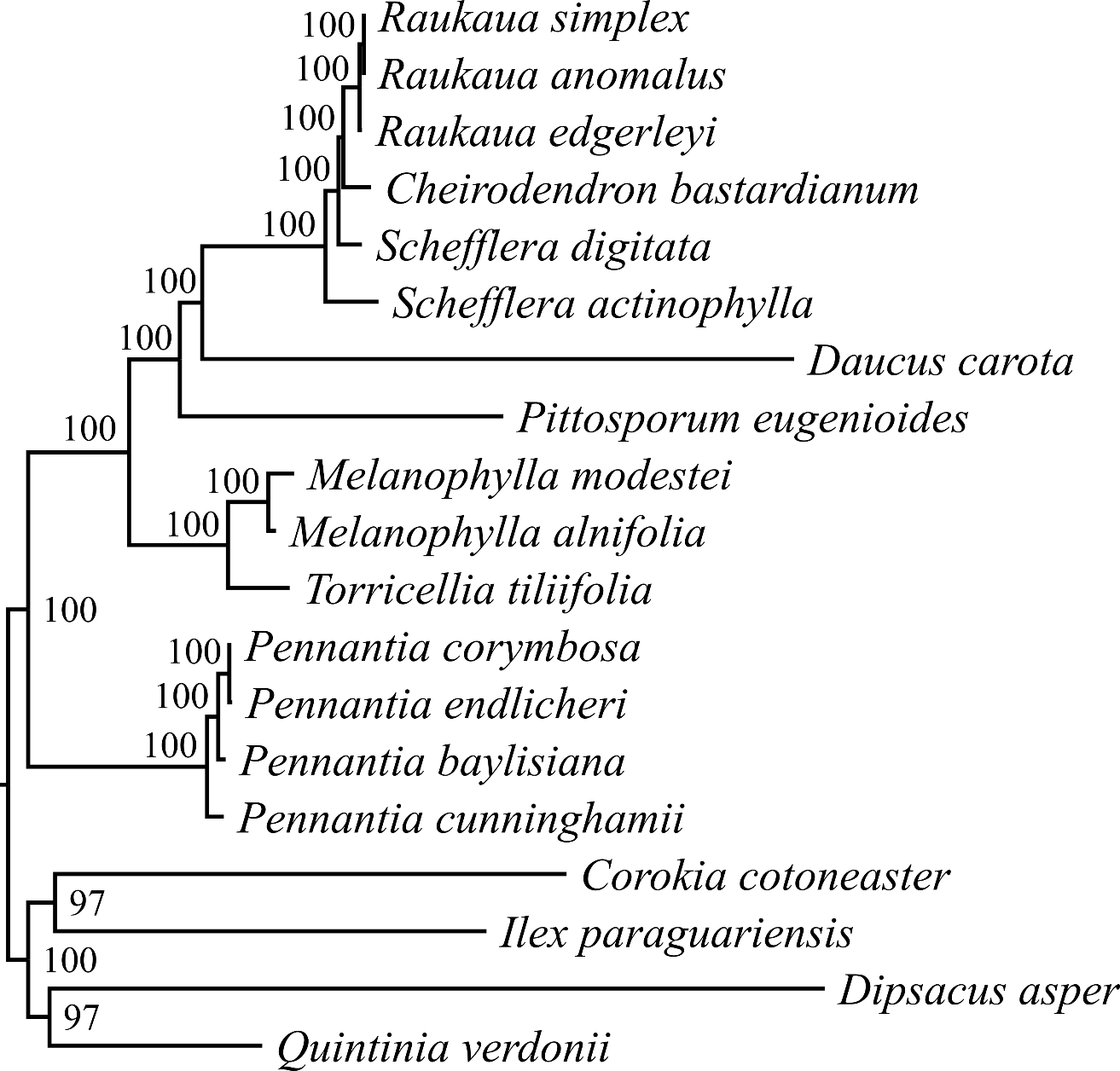 |
| --- |

**Supplementary Figure 4.** Undated 18S–26S nuclear DNA repeat region BEAST 2 phylogeny of *Pennantia*, under the Yule model. The tree was rooted to make *P. cunninghamii* sister to the other species of *Pennantia*, in accordance with the chloroplast DNA tree and the ITS tree of Keeling et al. (2004). Node posterior probability is shown next to the corresponding node. The sequences downloaded from GenBank have their accession number in round brackets; the others were generated from the samples used in this study.

| 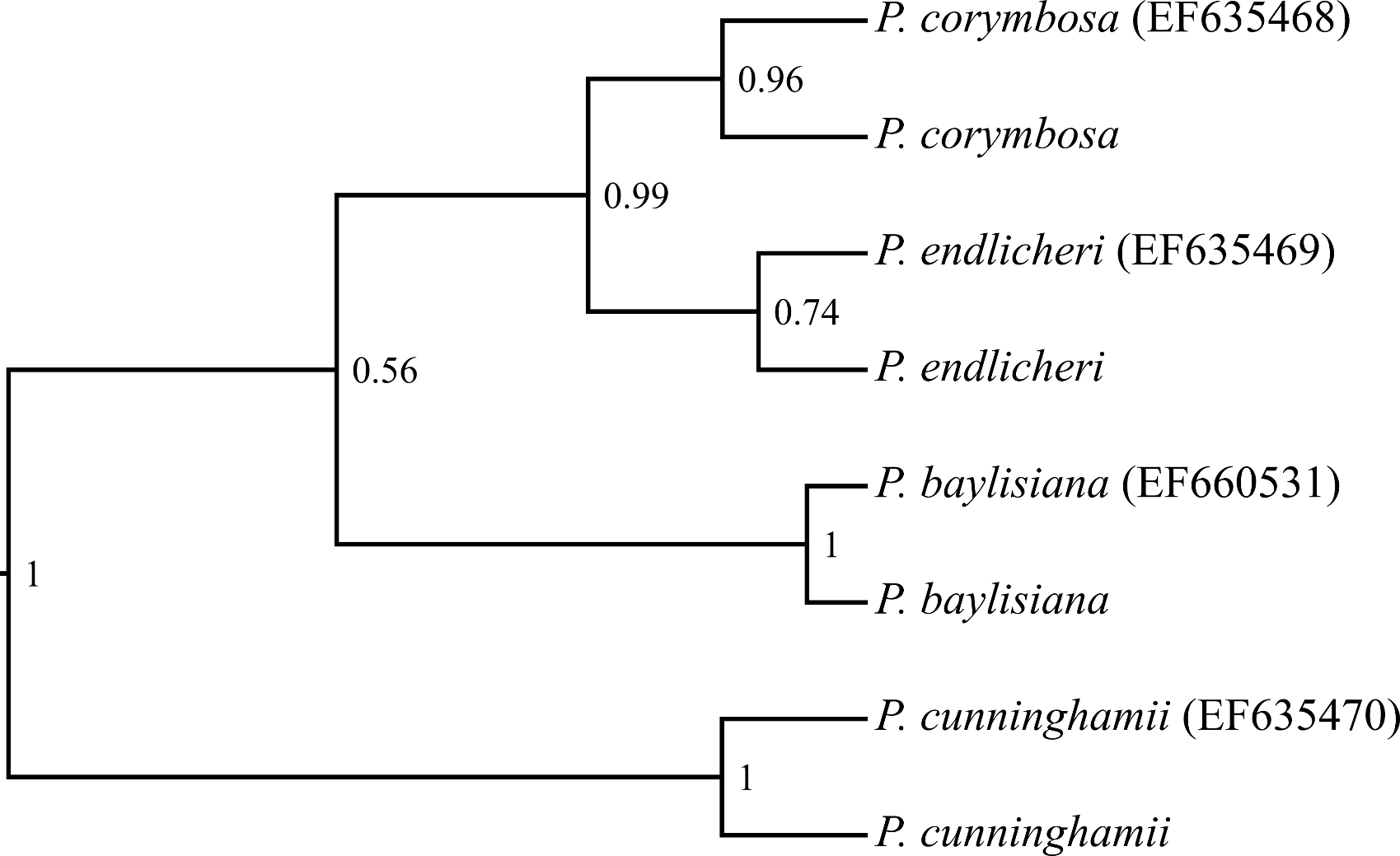 |
| --- |

**Supplementary Figure 5.** RAxML reconstruction of the 18S–26S nuclear DNA repeat region phylogeny of *Pennantia*. The tree was rooted to make *P. cunninghamii* sister to the other species of *Pennantia*, in accordance with the chloroplast DNA tree of this study and the ITS tree of Keeling et al. (2004). Bootstrap value is indicated next to the corresponding node.

| 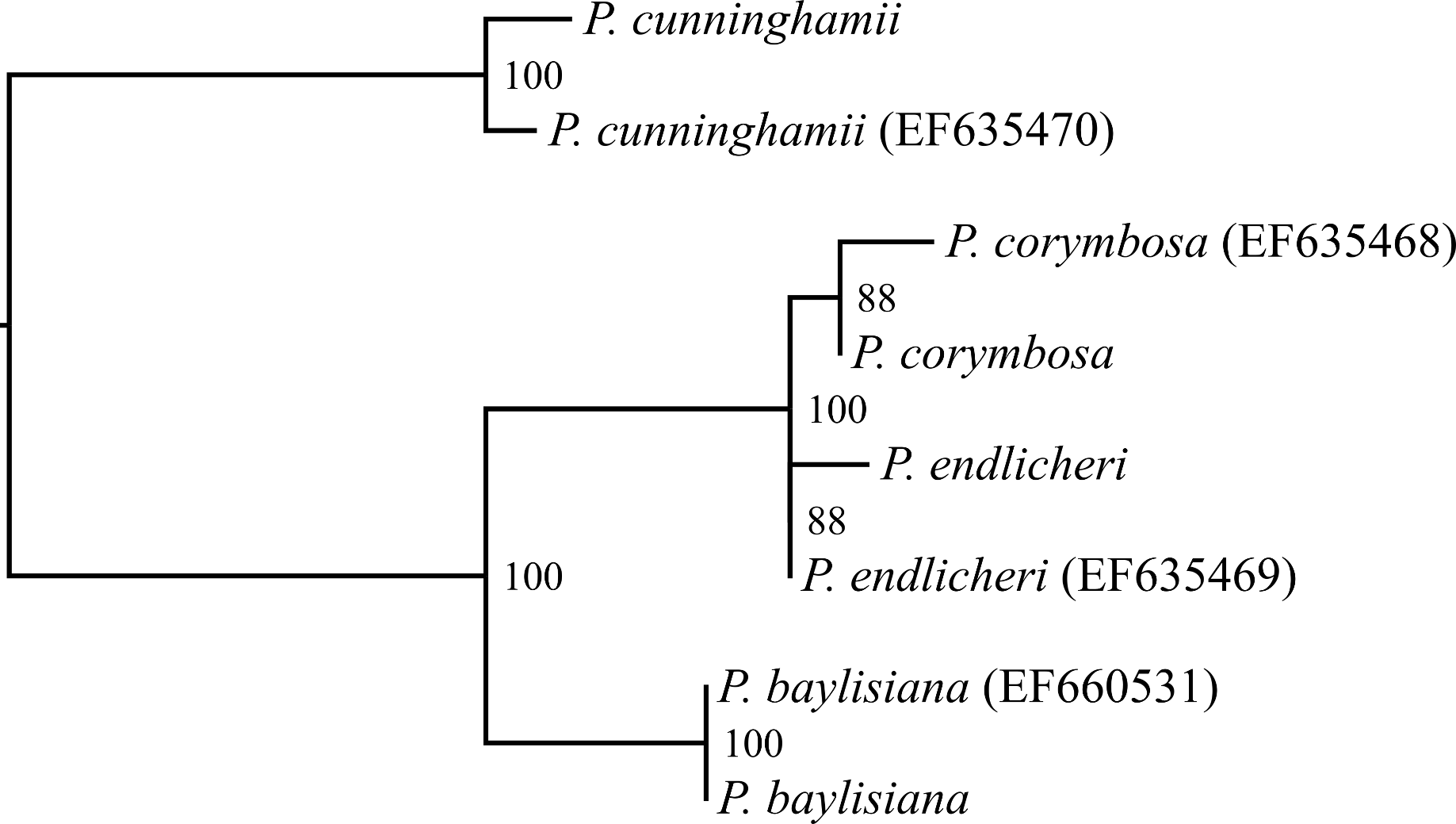 |
| --- |

**Supplementary Table 1.** Statistics on the quality before annotation of the chloroplast DNA sequences generated from herbarium samples.

| **Species** | **Herbarium accession #** | **% of ambiguities in sequence** | **HQ%** | **Mean coverage** |
| --- | --- | --- | --- | --- |
| *Cheirodendron bastardianum* (Decne.) Frodin | P02800554 | 0.2% | 99.9 | 1,905.3 |
| *Corokia cotoneaster* Raoul | CHR 655097 | 6.2% | 93.7 | 10,139.9 |
| *Melanophylla alnifolia* Baker | P02529054 | 1.0% | 99.1 | 812.8 |
| *Melanophylla modestei* G.E. Schatz, Lowry & A.-E. Wolf | P06233571 | 4.8% | 63.6 | 16.5 |
| *Pennantia baylisiana* (W.R.B.Oliv.) G.T.S.Baylis | CHR 655088 | 0.5% | 99.5 | 10,296.6 |
| *Pennantia corymbosa* J.R.Forst. & G.Forst. | CHR 649661 | 0.8% | 97.7 | 176.2 |
| *Pennantia cunninghamii* Miers | CANB869762 | 0.4% | 99.5 | 8,342.9 |
| *Pennantia endlicheri* Reissek | CBG8703383 | 0.5% | 99.5 | 2,523.0 |
| *Pittosporum eugenioides* A.Cunn. | CHR 553618 | 2.0% | 98.2 | 10,803.7 |
| *Raukaua anomalus* (Hook.) A.D.Mitch., Frodin & Heads | CHR 649673 | 0.3% | 99.5 | 456.8 |
| *Raukaua edgerleyi* (Hook.f.) Seem. | CHR 655508 | 0.2% | 99.9 | 1,536.7 |
| *Raukaua simplex* (G.Forst.) A.D.Mitch., Frodin & Heads | CHR 437312 | 0.2% | 99.9 | 1,624.3 |
| *Schefflera digitata* J.R.Forst. & G.Forst. | CHR 649676 | 2.1% | 94.0 | 123.6 |

**Supplementary Table 2.** Statistics on the quality before annotation of the 18S–26S nuclear DNA repeat region sequences generated from herbarium samples.

| **Species** | **Herbarium accession #** | **# of reads unmapped to chloroplast DNA reference** | **# of reads mapped to chimeric ITS region reference** | **% of ambiguities in sequence** | **HQ%** | **Mean coverage** |
| --- | --- | --- | --- | --- | --- | --- |
| *Melanophylla alnifolia* Baker | P02529054 | 73,154 | 9 | 93.9% | 5.0 | 0.2 |
| *Melanophylla modestei* G.E. Schatz, Lowry & A.-E. Wolf | P06233571 | 9,142 | 10 | - | 100.0 | 0.2 |
| *Pennantia baylisiana* (W.R.B.Oliv.) G.T.S.Baylis | CHR 655088 | 872,406 | 6,784 | 0.0% | 100.0 | 144.5 |
| *Pennantia corymbosa* J.R.Forst. & G.Forst. | CHR 649661 | 39,486 | 97 | 29.1% | 51.0 | 2.0 |
| *Pennantia cunninghamii* Miers | CANB869762 | 1,482,746 | 1,512 | 1.9% | 98.0 | 28.0 |
| *Pennantia endlicheri* Reissek | CBG8703383 | 383,410 | 411 | 6.7% | 86.2 | 8.7 |

**Supplementary Table 3.** List of the 60 protein-coding sequences used in the chloroplast DNA analyses.

| atpA | petG | psbN |
| --- | --- | --- |
| atpB | petL | psbT |
| atpE | petN | psbZ |
| atpF | psaA | rbcL |
| atpH | psaB | rpl14 |
| atpI | psaC | rpl16 |
| cemA | psaI | rpoA |
| matK | psaJ | rpoB |
| ndhA | psbA | rpoC1 |
| ndhC | psbB | rpoC2 |
| ndhE | psbC | rps11 |
| ndhF | psbD | rps14 |
| ndhG | psbE | rps15 |
| ndhH | psbF | rps16 |
| ndhI | psbH | rps2 |
| ndhJ | psbI | rps3 |
| ndhK | psbJ | rps4 |
| petA | psbK | rps8 |
| petB | psbL | ycf3 |
| petD | psbM | ycf4 |
